# Supplementary material for: MSClustering: A Cytoscape Tool for Multi-Level Clustering of Biological Networks
Source: Int J Mol Sci. 2022 Nov 17;23(22):14240. doi: 10.3390/ijms232214240 (PMC9699063; doi:10.3390/ijms232214240)
Supplement: Supplementary file 1 [file ijms-23-14240-s001.zip › ijms-1992181-supplementary.pdf]

Supplementary Information

For **“MSClustering: A Cytoscape tool for multi-level clustering of biological networks”** by BK GE, GM Hu, RHG Chen, and CM Chen

Table of Contents

I. Supplementary Results (pages 1-3).

II. Materials and Methods (pages 4-23).

III. Manual of the MSClustering app (pages 24-28).

I. Supplementary Results

Figure S1 demonstrates the validity of MSClustering in clustering large systems (3481 enzyme sequences). In Figure S1, MSClustering divides these enzymes into 7 groups, each corresponding to an EC category. The results of MSClustering are mostly consistent with the EC classification. Compared with state-of-the-art methods, MSClustering is efficient and reliable for clustering large systems. In addition, MSClustering is robust under monotonic transformations of the defined distance between nodes. In Figure S2, we show a drastic change in the clustering results of 197 GPCRs by using the neighbor-joining method for the distance to be (A) $E^{0.02}$ or (B) E.

Table S1 gives a comparison of MSClustering with the five most popular clustering plugins in Cytoscape.

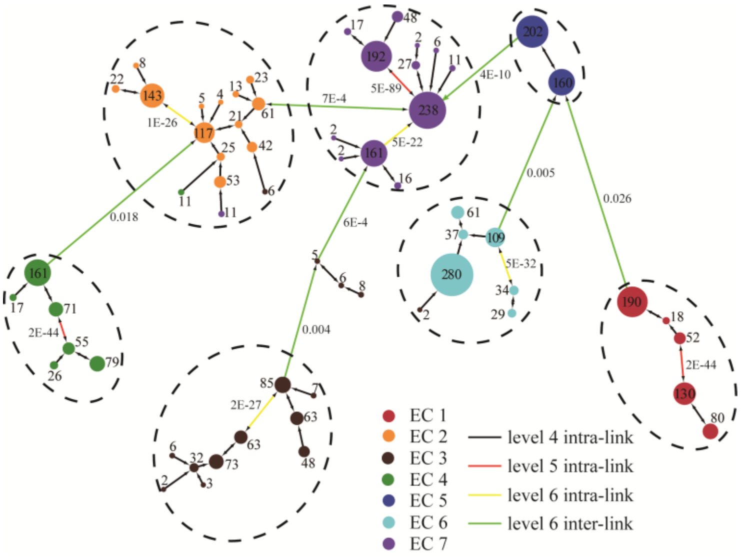

Figure S1. A distance-based phylogenetic tree of the enzyme network by MSClustering. The clustering structure of the network is shown for MSC levels 4-6. Each node is a

level 3 cluster and is colored according to the EC classification of its members. The number on the nodes denotes the number of enzymes in the cluster. The number on the edges between nodes is the smallest *E* value between clusters.

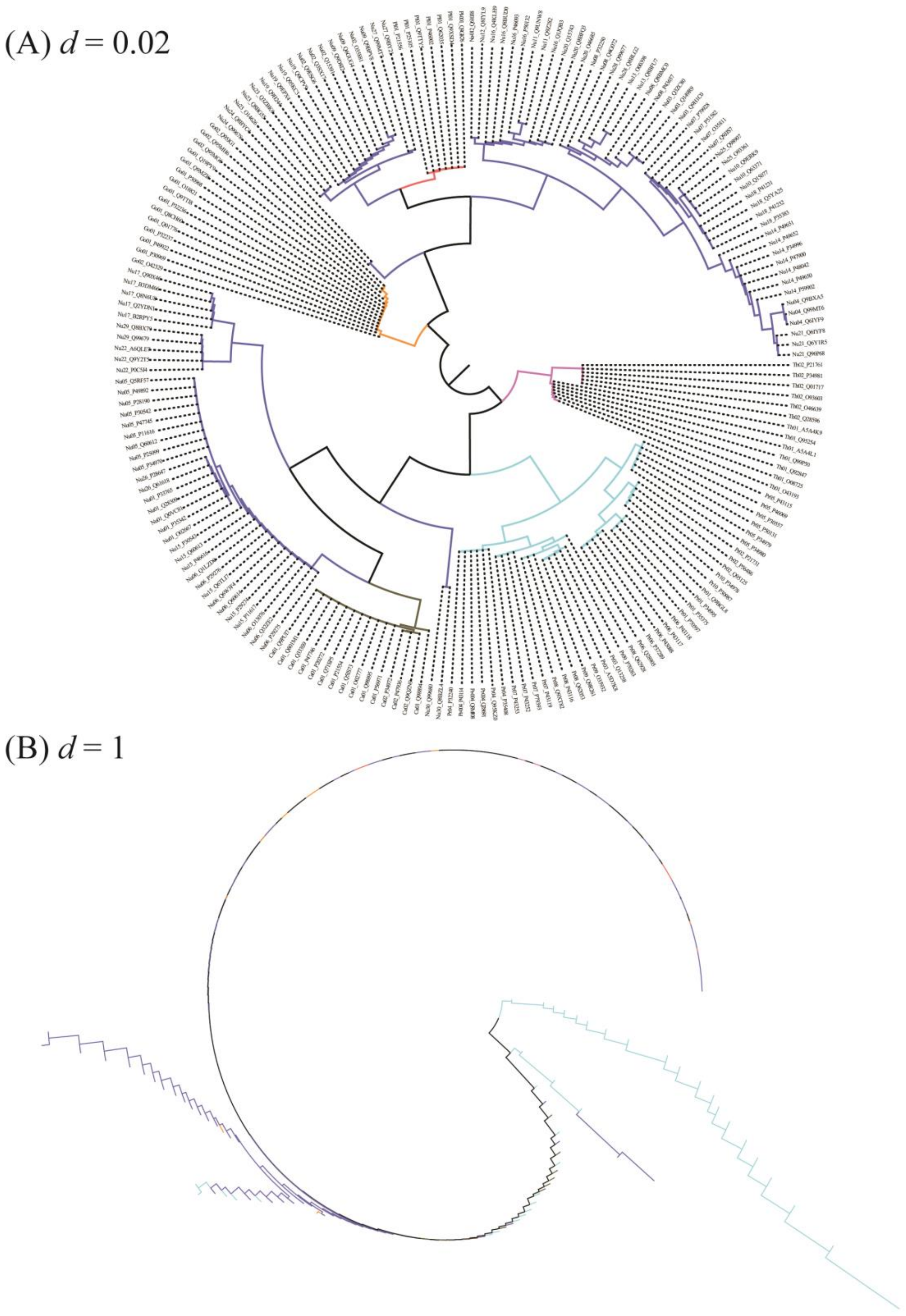

Figure S2. Distance-based phylogenetic trees (polar layout) of the GPCR network constructed by N-J using the distance defined as $E^{0.02}$ (A) or E (B), where E is the BLAST E value.

Table S1 Comparison of the five most popular clustering plugins in Cytoscape with MSClustering. The download statistics was generated on 2022/11/12.

**Plugin**	**Features**	**Input**	**Clustering Algorithms**	**Downloads**	**Average Rating (# of Ratings)**
AutoAnnotate	finds clusters and visually annotates them with labels and groups	selected Cytoscape network	MCL[defaulat], Affinity Propagation (AP), Cluster Fuzzifier (CF), Community clustering (CC), Connected Components Clustering (CCC), Spectral Clustering of Protein Sequences (SCPS)	43997	5/5 (37)
clusterMaker2	unifies a variety of algorithms for clustering networks and attributes as well as for ranking clusters based on potentially orthogonal data	selected Cytoscape network	Attribute Clustering:<br>AutoSOME Clustering,<br>Hierarchical Clustering,<br>K-Means Clustering,<br>K-Medoid Clustering,<br>HOPACH Clustering,<br>Partition Around Medoids (PAM) Clustering<br><br>Network Clustering:<br>AP, CF, CCC, CC, Fuzzy C-Means Clustering, MCODE, MCL, SCPS, Leiden Clustering, Transitivity Clustering, Infomap, Fast Greedy, Leading Eigenvector, Label Propagation, Multilevel Clustering	17618	4/5 (23)
ClusterViz	found cluster can be subjected to GO enrichment analysis.	selected Cytoscape network	FAG-EC, MCODE, EAGLE	20094	4.1/5 (13)
CytoCluster	analyzes and visualizes clusters from network	selected Cytoscape network	HC-PIN, DCU, OH-PIN, IPCA, IPC-MCE, ClusterONE	29568	5/5 (12)
MCODE	clusters a given network based on topology to find densely connected regions	selected Cytoscape network	MCODE	141356	4.5/5 (30)
MSClustering	an efficient app for hierarchical clustering and phylogenetics of complex networks	distance matrix	Minimum span clustering (MSC)	1171	5/5 (26)
Link:<br>https://apps.cytoscape.org/apps/autoannotate; https://apps.cytoscape.org/apps/clustermaker2;<br>https://apps.cytoscape.org/apps/clusterviz; https://apps.cytoscape.org/apps/cytocluster;<br>https://apps.cytoscape.org/apps/mcode; https://apps.cytoscape.org/apps/msclustering;					

II. Materials and Methods

The MSClustering app is an easy-to-use Cytoscape tool designed to visualize the clustering and phylogenetic information of complex systems. For demonstration, we first applied it to cluster 63 beta coronaviruses (obtained from GISAID and NCBI GenBank, and listed in Table S2) that were previously studied by Jaimes et al. Since amino acid sequences of their S protein were used to calculate the mutual distances between viruses, viruses with identical S protein sequence will have zero distance and be simplified as one node. In total, we have 46 nodes as listed in Table S2.

The alignment of 46 coronaviruses was performed on their S protein sequences using the MAFFT version7. The character-based phylogenetic tree was generated by the best model found with IQ-TREE using maximum-likelihood estimation. Table S3 lists the scores of two popular information criteria, including AIC (Akaike's information criterion) and BIC (Baysian Information Criterion). Both criteria suggest that, for this case, the WAG (+F, empirical AA frequencies; +R4, default free-rate heterogeneity) model is the best empirical model using maximal likelihood.

Furthermore, we used MSClustering to investigate the phylogenetics of 197 GPCRs as listed in Table S4. In this case study, we used three distance-based methods (MSClustering, N-J, and Louvain) and three character-based methods (IQ-TREE, PhyML, and ProtTest3) to construct the phylogenetic trees. For distance-based methods, we used the BLAST E value or $E^{0.02}$ to denote the distance between GPCRs. The results of MSClustering at various characteristic levels are shown in Table S4. Table S5 shows the comparison of GPCRs’ clustering results (47 clusters) obtained from MSClustering, N-J, and Louvain.

The MSClustering app was developed based on the MSC algorithm that has been previously used to study various complex networks in biology, literature, finance, and scientometrics. The flow chart of MSC is shown in Figure S3. As a Cytoscape tool, it is freely available and can be used to visualize the hierarchical clustering and phylogenetic information of complex systems. Here, as in the flowchart of Figure S4, we briefly describe the four steps in the MSClustering:

Step 1 (simplification). The MSC algorithm identifies the closest neighbor of each node and records their distances in a list of ascending order from the distance matrix. For a

network of N nodes, instead of dealing with a distance matrix of N^2 elements, MSC only processes at most N distances in the list.

Step 2 (clustering). MSC constructs the first cluster by starting from the shortest node pair and then including additional pairs from the list in the order of increasing distance. For the added distance, if one of the two nodes is involved in one of the constructed clusters, the size of this cluster increases but the number of clusters remains the same. If both nodes of the distance are not involved in the above-constructed clusters, a new cluster is identified, and the number of clusters increases. All clusters of the network are found when all distances in the list are considered. The identified clusters in the first run are referred to as the first level clustering, which has the finest resolution.

Step 3 (renormalization). Clusters constructed in step 2 are considered renormalized nodes, and the distance between clusters is calculated by finding the shortest distance of inter-cluster node pairs between two clusters. The network consisting of these renormalized components is further clustered by steps 1-2, and higher levels of clustering with a lower resolution are constructed. Steps 1 and 2 are repeated until the number of groups is smaller than N_{limit}.

Step 4 (outlier detection). For high-level (late-stage) clustering, the number of renormalized nodes is small and the consistency of predicted clusters is reduced due to the inclusion of outliers. A renormalized node is considered an outlier if its shortest distance to other nodes is larger than $m \times L_{\text{med}}$, where m is the threshold parameter and L_{med} is the median of the shortest distance list. MSClustering will detect outliers for the last two levels of clustering, and each outlier forms a cluster by itself.

Figure S5 is a demonstration of using MSClustering to visualize the hierarchical clustering of the beta coronavirus network at various levels of resolution (A) and its distance-based phylogenetic tree (B).

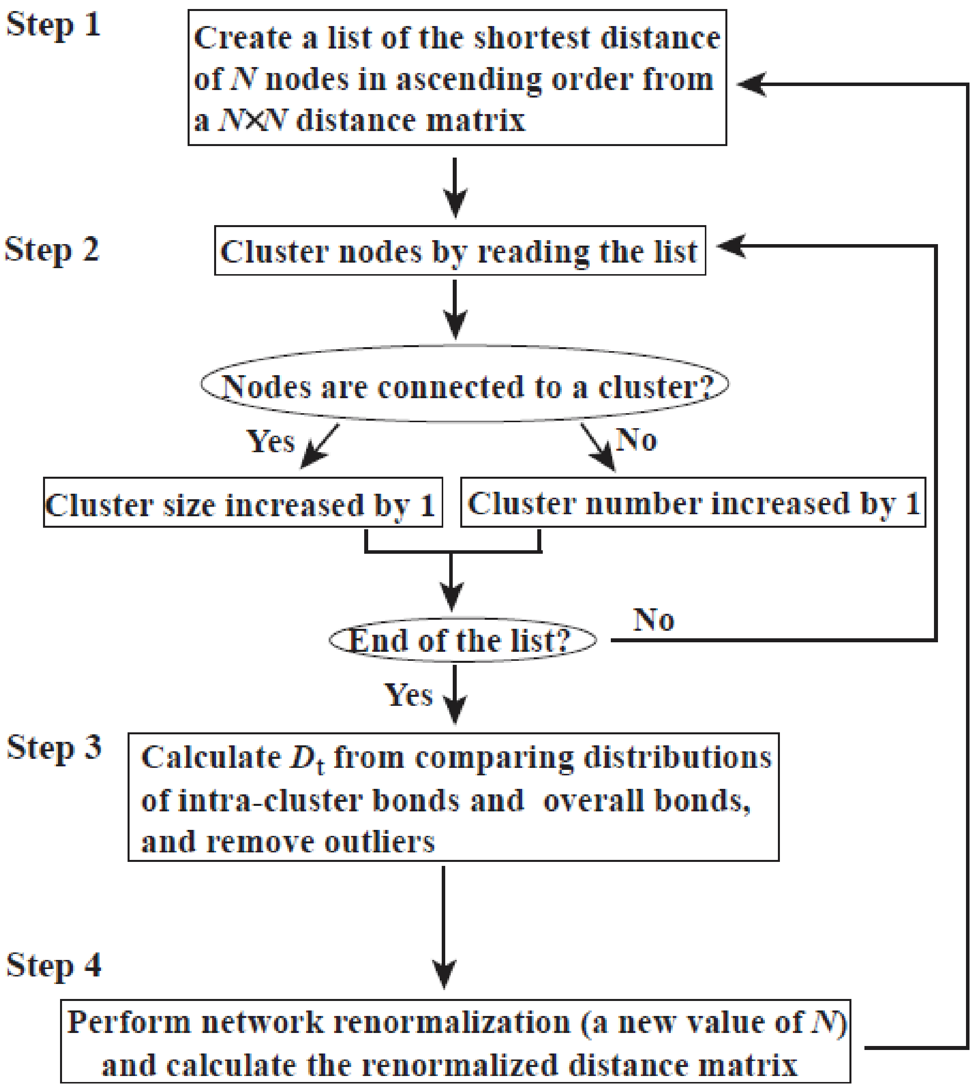

Figure S3. Flowchart of the MSC algorithm.

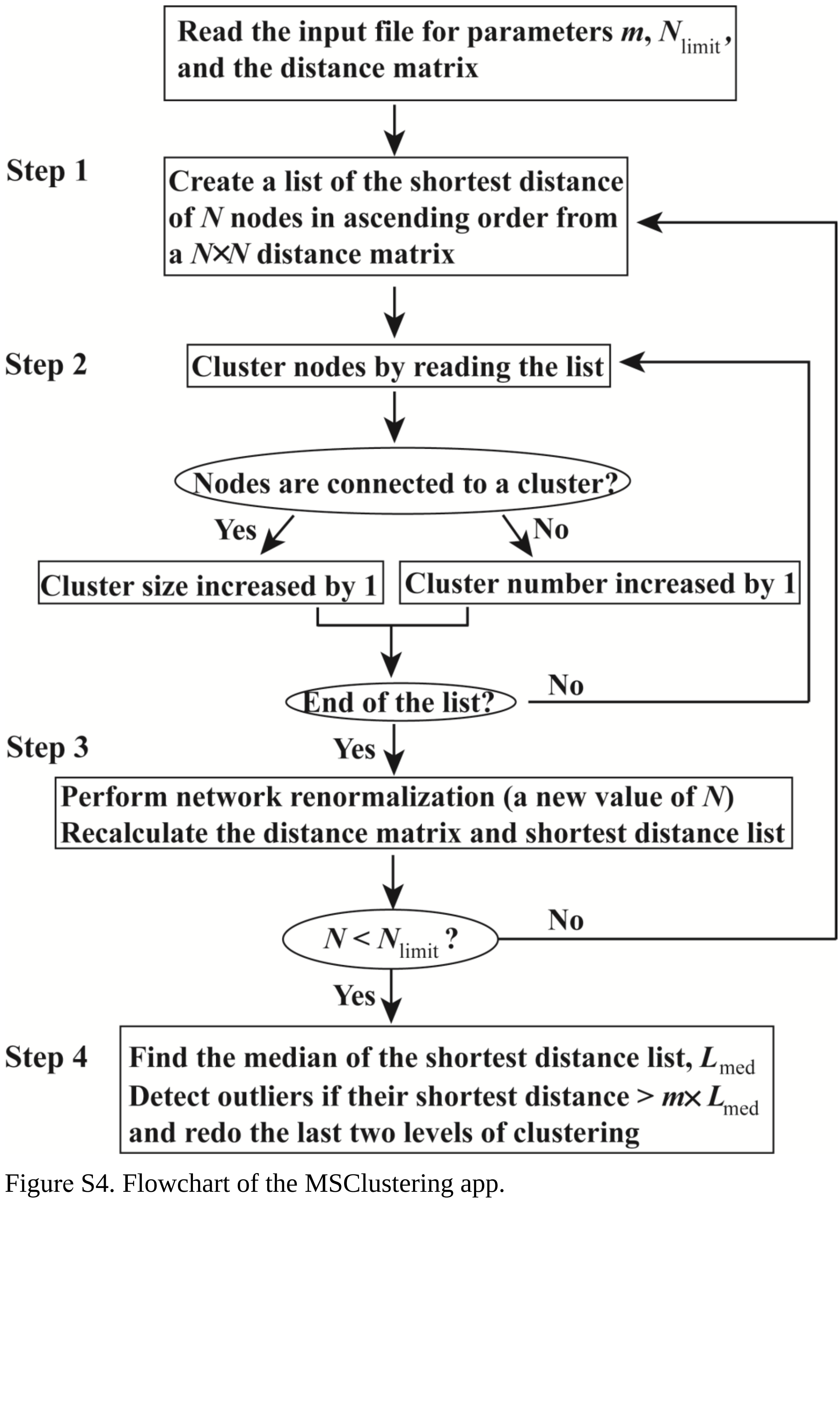

Figure S4. Flowchart of the MSClustering app.

(A)

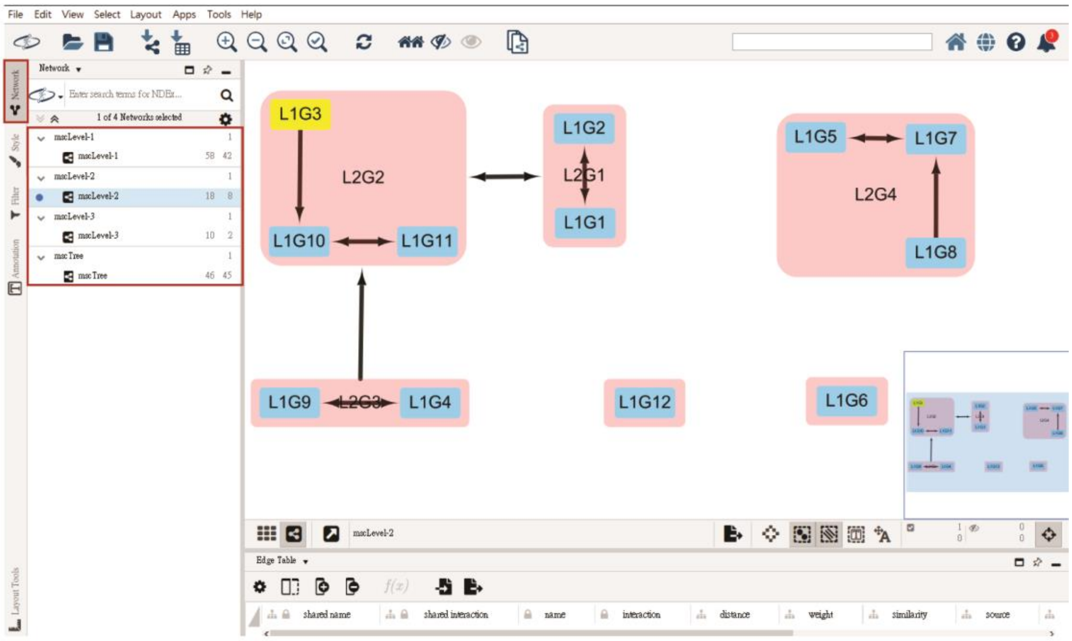

(B)

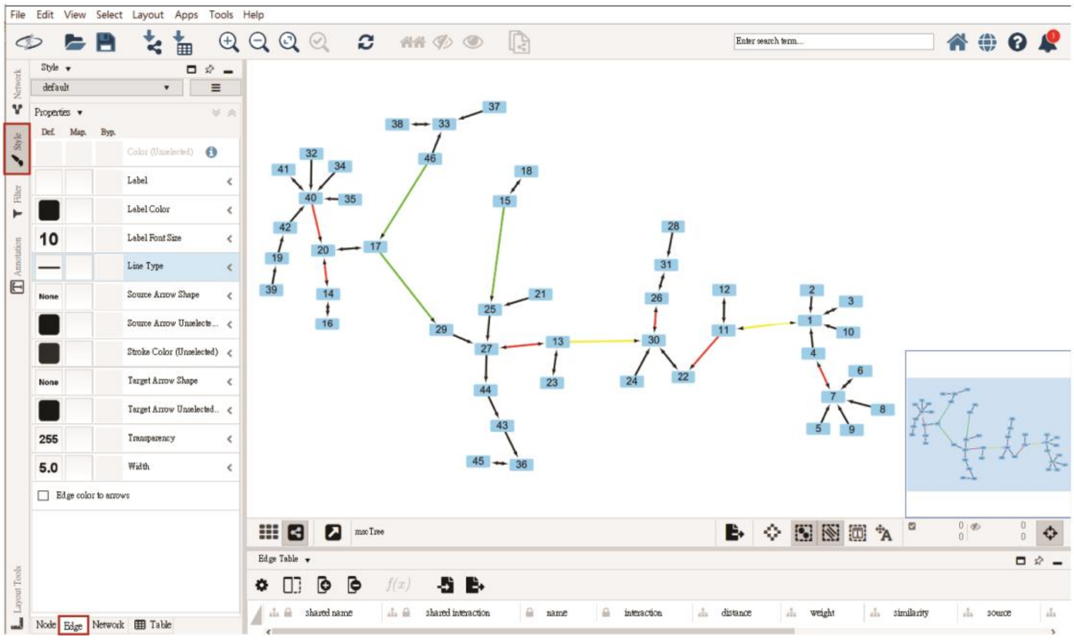

Figure S5. Demonstration of MSClustering for the multi-level clustering (A) and the MSC tree (B). We show the Network panel in (A) and the Style panel in (B).

Table S2. 63 betacoronavirus sequences in the target dataset. Betacoronaviruses with the same S protein sequence are colored in red and labeled by the same node ID.

Sequence label	Accession ID	Node ID
SARS-CoV-2-Foshan	EPI_ISL_406535	1
SARS-CoV-2-Foshan	EPI_ISL_406534	1
SARS-CoV-2-Guangdong	EPI_ISL_406538	1
SARS-CoV-2-Guangzhou	EPI_ISL_406533	1
SARS-CoV-2-Nonthaburi	EPI_ISL_403962	1
SARS-CoV-2-Shenzhen	EPI_ISL_405839	1
SARS-CoV-2-Taiwan	EPI_ISL_406031	1
SARS-CoV-2-USA-CA1	EPI_ISL_406034	1
SARS-CoV-2-USA-WA1	EPI_ISL_404895	1
SARS-CoV-2-Wuhan-WIV06	EPI_ISL_402129	1
SARS-CoV-2-Zhejiang	EPI_ISL_404228	1
BetaCoV/Wuhan/IVDC-HB-envF13–20/2020	EPI_ISL_408514	1
BetaCoV/Wuhan/IVDC-HB-envF13–21/2020	EPI_ISL_408515	1
SARS-CoV-2-Wuhan-Hu1	MN908947.3	1
SARS-CoV-2-France	EPI_ISL_406596	2
SARS-CoV-2-USA-IL1	EPI_ISL_404253	3
Bat-SL-RaTG13	EPI_ISL_402131	4
BetaCoV/pangolin/Guangxi/P5E/2017	EPI_ISL_410541	5
Beta-CoV/pangolin/Guangxi/P2V/2017	EPI_ISL_410542	6
BetaCoV/pangolin/Guangxi/P5L/2017	EPI_ISL_410540	7
Beta-CoV/pangolin/Guangxi/P4L/2017	EPI_ISL_410538	8
BetaCoV/pangolin/Guangxi/P1E/2017	EPI_ISL_410539	9
BetaCoV/Wuhan/IVDC-HB-envF13/2020	EPI_ISL_408511	10
Bat-SL-CoVZC45	MG772933.1	11
Bat-SL-CoVZXC21	MG772934.1	12
Bat-SL-LYRa3	KF569997.1	13
BatCoV/133	DQ648794.1	14
BatCoV-GCCDC1	NC_030886.1	15
BatCoV-HKU4–1	EF065505.1	16
BatCoV-HKU5–1	EF065509.1	17
BatCoV-HKU9	NC_009021.1	18
BatCoV-Neo/PML-PHE1/RSA	KC869678.4	19
BtVs-BetaCoV/SC2013	KJ473821.1	20
Bat-CoV-BM48–31	NC_014470.1	21
Bat-SL-HKU3–1	DQ022305.2	22
Bat-SL-LYRa11	KF569996.1	23
Bat-SL-Rf1	DQ412042.1	24
Bat-SL-Rs4231	KY417146.1	25

Bat-SL-Rs4255	KY417149.1	26
Bat-SL-Rs4874	KY417150.1	27
Bat-SL-RS672	FJ588686.1	28
Bat-SL-WIV1	KC881007.1	29
BtRs-BetaCoV/YN2018C	MK211377.1	30
BtRs-BetaCoV/YN2018D	MK211378.1	31
camMERS-CoV-HKFU-HKU-13	KJ650295.1	32
camMERS-CoV-HKU23	KF906251.1	33
camMERS-CoV-KSA-505	KJ713295.1	33
amMERS-CoV-NRCE-HKU205	KJ477102.1	34
camMERS-CoV-NRCE-HKU270	KJ477103.2	35
CivSARS-CoV-SZ3	P59594.1	36
hSARS-CoVHKU39849	JN854286.1	36
HCoV-HKU1	AY597011.2	37
HCoV-OC43	KF963244.1	38
Hedgehog-CoV/VMC/DEU	KC545383.1	39
hMERSCoV-EMC/2012	JX869059.2	40
hMERS-CoV-England-1	KC164505.2	41
hMERS-CoV-Jordan-N3	KC776174.1	42
hSARS-CoV-BJ01	AY278488.2	43
hSARS-CoV-GZ02	AY390556.1	44
hSARS-CoV-Tor2	NC_004718.3	45
MHV-A59	M18379.1	46

Table S3. Scores of AIC and BIC for the phylogenetic tree of the system constructed by various evolution models.

**Model**	**AIC**	**BIC**
WAG+F+R4	72139.45	72750.28
LG+F+R4	72329.65	72940.48
VT+F+R4	72331.02	72941.85
Blosum62+F+R4	72454.11	73064.94
PMB+F+R4	72496.5	73107.33
WAG+R4	72626.63	73135.66
VT+R4	72672.69	73181.72
JTTDCMut+F+R4	72621.21	73232.05
JTT+F+R4	72630.68	73241.52
rtREV+F+R4	72651.74	73262.57
LG+R4	72757.33	73266.36
LG+R5	72760.68	73280.42
LG+I+G4	72793.37	73280.96
LG+G4	72825.56	73307.8
LG+R3	72840.31	73338.62
DCMut+F+R4	72737.88	73348.72
Dayhoff+F+R4	72742.58	73353.42
cpREV+F+R4	72833.28	73444.11
PMB+R4	73000.55	73509.58
FLU+F+R4	72908.2	73519.04
JTTDCMut+R4	73022.78	73531.81
JTT+R4	73037.16	73546.19
Blosum62+R4	73048.55	73557.58
cpREV+R4	73199.37	73708.4
LG+R2	73258.75	73746.35
mtInv+F+R4	73234.3	73845.13
rtREV+R4	73401.73	73910.76
FLU+R4	73420.91	73929.93
DCMut+R4	73478.3	73987.33
Dayhoff+R4	73483.11	73992.14
mtREV+F+R4	73564.96	74175.8
mtMet+F+R4	73733	74343.84
mtZOA+F+R4	73752.31	74363.15
HIVb+F+R4	74082.96	74693.8
LG+I	74406.65	74888.88
mtVer+F+R4	74482.15	75092.99
mtART+F+R4	74560.09	75170.93
HIVb+R4	74918.79	75427.82

mtMAM+F+R4	75011.49	75622.33
HIVw+F+R4	75059.14	75669.98
mtZOA+R4	75419.35	75928.38
LG	75454.13	75931.01
mtMet+R4	75609.72	76118.75
mtREV+R4	75652.16	76161.19
mtInv+R4	75849.55	76358.58
mtVer+R4	76398.3	76907.32
HIVw+R4	76520.32	77029.34
mtART+R4	76550.43	77059.46
mtMAM+R4	77340.9	77849.93

Table S4. Details of 197 GPCR sequences and their multi-level MSC clustering. Here protein type is obtained from GPCRdb and protein information is obtained from UniProtKB.

MSC 4	MSC 2	MSC 1	Uniprot ID	Protein type	protein information
I	1	Pl01	P21556	Platelet-activating factor	Platelet-activating factor receptor
I	1	Pl01	Q9TTY5	Platelet-activating factor	Platelet-activating factor receptor
I	1	Pl01	Q62035	Platelet-activating factor	Platelet-activating factor receptor
I	1	Pl01	Q9XSD4	Platelet-activating factor	Platelet-activating factor receptor
I	1	Pl01	P25105	Platelet-activating factor	Platelet-activating factor receptor
I	1	Pl01	Q9GK76	Platelet-activating factor	Platelet-activating factor receptor
I	1	Pl01	P46002	Platelet-activating factor	Platelet-activating factor receptor
I	1	Nu08	Q8BMC0	Nucleotide-like	P2Y5/LPA6
I	1	Nu08	P43657	Nucleotide-like	P2Y5/LPA6
I	1	Nu08	Q4G072	Nucleotide-like	P2Y5/LPA6
I	1	Nu08	P32250	Nucleotide-like	P2Y5/LPA6
I	1	Nu03	Q149R9	Nucleotide-like	GPR92/LPA5
I	1	Nu03	Q3ZC80	Nucleotide-like	GPR92/LPA5
I	1	Nu03	Q9H1C0	Nucleotide-like	GPR92/LPA5
I	1	Nu28	Q8BLG2	Nucleotide-like	P2Y9/LPA4
I	1	Nu28	Q99677	Nucleotide-like	P2Y9/LPA4
I	1	Nu13	O00398	Nucleotide-like	P2Y10
I	1	Nu13	Q8BFU7	Nucleotide-like	P2Y10
I	1	Nu24	Q99678	Nucleotide-like	GPR20
I	1	Nu24	Q8BYC4	Nucleotide-like	GPR20
I	2	Nu19	Q9CPV9	Nucleotide-like	P2Y12
I	2	Nu19	Q9H244	Nucleotide-like	P2Y12
I	2	Nu19	Q9EPX4	Nucleotide-like	P2Y12
I	2	Nu19	Q95KC3	Nucleotide-like	P2Y12
I	2	Nu02	Q9ESG6	Nucleotide-like	P2Y14
I	2	Nu02	O35881	Nucleotide-like	P2Y14
I	2	Nu02	Q3SX17	Nucleotide-like	P2Y14
I	2	Nu02	Q15391	Nucleotide-like	P2Y14
I	2	Nu09	Q9D8I2	Nucleotide-like	P2Y13
I	2	Nu09	Q6GUG4	Nucleotide-like	P2Y13
I	2	Nu09	Q9BPV8	Nucleotide-like	P2Y13
I	2	Nu23	Q8BG55	Nucleotide-like	GPR171
I	2	Nu23	Q3ZBK9	Nucleotide-like	GPR171
I	2	Nu23	O14626	Nucleotide-like	GPR171
I	2	Nu27	Q99MT7	Nucleotide-like	GPR87
I	2	Nu27	Q9BY21	Nucleotide-like	GPR87
I	3	Nu14	P49651	Nucleotide-like	P2Y1

I	3	Nu14	P34996	Nucleotide-like	P2Y1
I	3	Nu14	P49650	Nucleotide-like	P2Y1
I	3	Nu14	P49652	Nucleotide-like	P2Y1
I	3	Nu14	P47900	Nucleotide-like	P2Y1
I	3	Nu14	P48042	Nucleotide-like	P2Y1
I	3	Nu14	P59902	Nucleotide-like	P2Y1
I	3	Nu07	Q9JJS7	Nucleotide-like	P2Y4
I	3	Nu07	P51582	Nucleotide-like	P2Y4
I	3	Nu07	O35811	Nucleotide-like	P2Y4
I	3	Nu07	P79928	Nucleotide-like	P2Y4
I	3	Nu18	P41232	Nucleotide-like	P2Y2
I	3	Nu18	Q5YA25	Nucleotide-like	P2Y2
I	3	Nu18	P41231	Nucleotide-like	P2Y2
I	3	Nu18	P35383	Nucleotide-like	P2Y2
I	3	Nu04	Q99MT6	Nucleotide-like	Succinate receptors
I	3	Nu04	Q6IYF9	Nucleotide-like	Succinate receptors
I	3	Nu04	Q9BXA5	Nucleotide-like	Succinate receptors
I	3	Nu21	Q6IYF8	Nucleotide-like	2-oxoglutarate receptor 1
I	3	Nu21	Q6Y1R5	Nucleotide-like	2-oxoglutarate receptor 1
I	3	Nu21	Q96P68	Nucleotide-like	2-oxoglutarate receptor 1
I	4	Nu16	Q8BUD0	Nucleotide-like	GPR4
I	4	Nu16	Q4KLH9	Nucleotide-like	GPR4
I	4	Nu16	Q1JQB3	Nucleotide-like	GPR4
I	4	Nu16	P46093	Nucleotide-like	GPR4
I	4	Nu16	P50132	Nucleotide-like	GPR4
I	4	Nu20	Q8BFQ3	Nucleotide-like	Ovarian cancer GPCR 1
I	4	Nu20	O46685	Nucleotide-like	Ovarian cancer GPCR 1
I	4	Nu20	Q15743	Nucleotide-like	Ovarian cancer GPCR 1
I	4	Nu11	Q9UNW8	Nucleotide-like	GPR132
I	4	Nu11	Q9Z282	Nucleotide-like	GPR132
I	4	Nu12	Q61038	Nucleotide-like	Psychosine receptor
I	4	Nu12	Q8IYL9	Nucleotide-like	Psychosine receptor
I	12	Nu10	Q9ERK9	Nucleotide-like	P2Y6
I	12	Nu10	Q63371	Nucleotide-like	P2Y6
I	12	Nu10	Q15077	Nucleotide-like	P2Y6
I	12	Nu25	Q98907	Nucleotide-like	P2Y3
I	12	Nu25	O93361	Nucleotide-like	P2Y3
II	5	Nu05	P28190	Nucleotide-like	Adenosine receptor A1
II	5	Nu05	P11616	Nucleotide-like	Adenosine receptor A1
II	5	Nu05	P49892	Nucleotide-like	Adenosine receptor A1
II	5	Nu05	P34970	Nucleotide-like	Adenosine receptor A1

II	5	Nu05	P47745	Nucleotide-like	Adenosine receptor A1
II	5	Nu05	Q5RF57	Nucleotide-like	Adenosine receptor A1
II	5	Nu05	Q60612	Nucleotide-like	Adenosine receptor A1
II	5	Nu05	P30542	Nucleotide-like	Adenosine receptor A1
II	5	Nu05	P25099	Nucleotide-like	Adenosine receptor A1
II	5	Nu01	Q0VC81	Nucleotide-like	Adenosine receptor A3
II	5	Nu01	O02667	Nucleotide-like	Adenosine receptor A3
II	5	Nu01	P33765	Nucleotide-like	Adenosine receptor A3
II	5	Nu01	P35342	Nucleotide-like	Adenosine receptor A3
II	5	Nu01	Q28309	Nucleotide-like	Adenosine receptor A3
II	5	Nu26	Q61618	Nucleotide-like	Adenosine receptor A3
II	5	Nu26	P28647	Nucleotide-like	Adenosine receptor A3
II	5	Nu30	Q8BZL4	Nucleotide-like	GPR22
II	5	Nu30	Q99680	Nucleotide-like	GPR22
II	8	Nu06	Q60614	Nucleotide-like	Adenosine receptor A2b
II	8	Nu06	P29276	Nucleotide-like	Adenosine receptor A2b
II	8	Nu06	P29275	Nucleotide-like	Adenosine receptor A2b
II	8	Nu06	Q32ZE2	Nucleotide-like	Adenosine receptor A2b
II	8	Nu06	Q1LZD0	Nucleotide-like	Adenosine receptor A2b
II	8	Nu06	O13076	Nucleotide-like	Adenosine receptor A2b
II	8	Nu06	Q6W3F4	Nucleotide-like	Adenosine receptor A2b
II	8	Nu15	P29274	Nucleotide-like	Adenosine receptor A2a
II	8	Nu15	P11617	Nucleotide-like	Adenosine receptor A2a
II	8	Nu15	P30543	Nucleotide-like	Adenosine receptor A2a
II	8	Nu15	Q60613	Nucleotide-like	Adenosine receptor A2a
II	8	Nu15	Q6TLI7	Nucleotide-like	Adenosine receptor A2a
II	8	Nu15	P46616	Nucleotide-like	Adenosine receptor A2a
II	8	Nu17	B3DM66	Nucleotide-like	GPR161
II	8	Nu17	B2RPY5	Nucleotide-like	GPR161
II	8	Nu17	Q90X46	Nucleotide-like	GPR161
II	8	Nu17	Q8N6U8	Nucleotide-like	GPR161
II	8	Nu17	Q2YDN1	Nucleotide-like	GPR161
III	6	Pr05	P43115	Prostanoid	Prostaglandin E2 receptor EP3
III	6	Pr05	P50131	Prostanoid	Prostaglandin E2 receptor EP3
III	6	Pr05	P46069	Prostanoid	Prostaglandin E2 receptor EP3
III	6	Pr05	P34979	Prostanoid	Prostaglandin E2 receptor EP3
III	6	Pr05	P34980	Prostanoid	Prostaglandin E2 receptor EP3
III	6	Pr05	P30557	Prostanoid	Prostaglandin E2 receptor EP3
III	6	Pr06	P43088	Prostanoid	Prostaglandin F2-alpha receptor
III	6	Pr06	P37289	Prostanoid	Prostaglandin F2-alpha receptor
III	6	Pr06	P43117	Prostanoid	Prostaglandin F2-alpha receptor

III	6	Pr06	P43118	Prostanoid	Prostaglandin F2-alpha receptor
III	6	Pr06	Q28905	Prostanoid	Prostaglandin F2-alpha receptor
III	6	Pr01	P35375	Prostanoid	Prostaglandin E2 receptor EP1
III	6	Pr01	P34995	Prostanoid	Prostaglandin E2 receptor EP1
III	6	Pr01	Q9BGL8	Prostanoid	Prostaglandin E2 receptor EP1
III	6	Pr01	P70597	Prostanoid	Prostaglandin E2 receptor EP1
III	14	Pr02	Q95125	Prostanoid	Thromboxane A2 receptor
III	14	Pr02	P21731	Prostanoid	Thromboxane A2 receptor
III	14	Pr02	P56486	Prostanoid	Thromboxane A2 receptor
III	14	Pr10	P34978	Prostanoid	Thromboxane A2 receptor
III	14	Pr10	P30987	Prostanoid	Thromboxane A2 receptor
III	7	Pr04	P43114	Prostanoid	Prostaglandin E2 receptor EP4
III	7	Pr04	P32240	Prostanoid	Prostaglandin E2 receptor EP4
III	7	Pr04	Q95KZ0	Prostanoid	Prostaglandin E2 receptor EP4
III	7	Pr04	Q8MJ08	Prostanoid	Prostaglandin E2 receptor EP4
III	7	Pr04	P35408	Prostanoid	Prostaglandin E2 receptor EP4
III	7	Pr04	Q28691	Prostanoid	Prostaglandin E2 receptor EP4
III	7	Pr08	Q62928	Prostanoid	Prostaglandin E2 receptor EP2
III	7	Pr08	Q62053	Prostanoid	Prostaglandin E2 receptor EP2
III	7	Pr08	P43116	Prostanoid	Prostaglandin E2 receptor EP2
III	7	Pr08	Q9XT82	Prostanoid	Prostaglandin E2 receptor EP2
III	7	Pr07	P79393	Prostanoid	Prostaglandin I2 receptor
III	7	Pr07	P43119	Prostanoid	Prostaglandin I2 receptor
III	7	Pr07	P43252	Prostanoid	Prostaglandin I2 receptor
III	7	Pr07	P43253	Prostanoid	Prostaglandin I2 receptor
III	15	Pr09	O35932	Prostanoid	Prostaglandin D2 receptor
III	15	Pr09	Q9R261	Prostanoid	Prostaglandin D2 receptor
III	15	Pr09	P70263	Prostanoid	Prostaglandin D2 receptor
III	15	Pr03	A5D7K8	Prostanoid	Prostaglandin D2 receptor
III	15	Pr03	Q13258	Prostanoid	Prostaglandin D2 receptor
IV	9	Th01	O08725	Thyrotropin-releasing hormone and Secretagogue	Growth hormone secretagogue receptor type 1
IV	9	Th01	O43193	Thyrotropin-releasing hormone and Secretagogue	Growth hormone secretagogue receptor type 1
IV	9	Th01	Q99P50	Thyrotropin-releasing hormone and Secretagogue	Growth hormone secretagogue receptor type 1
IV	9	Th01	A5A4L1	Thyrotropin-releasing hormone and Secretagogue	Growth hormone secretagogue receptor type 1
IV	9	Th01	A5A4K9	Thyrotropin-releasing hormone and Secretagogue	Growth hormone secretagogue receptor type 1
IV	9	Th01	Q92847	Thyrotropin-releasing hormone and Secretagogue	Growth hormone secretagogue receptor type 1
IV	9	Th01	Q95254	Thyrotropin-releasing hormone and Secretagogue	Growth hormone secretagogue receptor type 1
IV	9	Th02	P21761	Thyrotropin-releasing hormone and Secretagogue	Thyrotropin-releasing hormone receptor

IV	9	Th02	Q01717	Thyrotropin-releasing hormone and Secretagogue	Thyrotropin-releasing hormone receptor
IV	9	Th02	O46639	Thyrotropin-releasing hormone and Secretagogue	Thyrotropin-releasing hormone receptor
IV	9	Th02	P34981	Thyrotropin-releasing hormone and Secretagogue	Thyrotropin-releasing hormone receptor
IV	9	Th02	O93603	Thyrotropin-releasing hormone and Secretagogue	Thyrotropin-releasing hormone receptor
IV	9	Th02	Q28596	Thyrotropin-releasing hormone and Secretagogue	Thyrotropin-releasing hormone receptor
V	10	Go01	Q01776	Gonadotropin-releasing hormone	Gonadotropin-releasing hormone I receptor
V	10	Go01	Q9MZI6	Gonadotropin-releasing hormone	Gonadotropin-releasing hormone I receptor
V	10	Go01	P30968	Gonadotropin-releasing hormone	Gonadotropin-releasing hormone I receptor
V	10	Go01	P32236	Gonadotropin-releasing hormone	Gonadotropin-releasing hormone I receptor
V	10	Go01	O18821	Gonadotropin-releasing hormone	Gonadotropin-releasing hormone I receptor
V	10	Go01	P32237	Gonadotropin-releasing hormone	Gonadotropin-releasing hormone I receptor
V	10	Go01	P49922	Gonadotropin-releasing hormone	Gonadotropin-releasing hormone I receptor
V	10	Go01	Q9TTI8	Gonadotropin-releasing hormone	Gonadotropin-releasing hormone I receptor
V	10	Go01	Q8CH60	Gonadotropin-releasing hormone	Gonadotropin-releasing hormone I receptor
V	10	Go01	Q19PY9	Gonadotropin-releasing hormone	Gonadotropin-releasing hormone I receptor
V	10	Go01	P30969	Gonadotropin-releasing hormone	Gonadotropin-releasing hormone I receptor
V	10	Go02	Q95MG6	Gonadotropin-releasing hormone	Gonadotropin-releasing hormone II receptor
V	10	Go02	Q95MH6	Gonadotropin-releasing hormone	Gonadotropin-releasing hormone II receptor
V	10	Go02	Q95JG1	Gonadotropin-releasing hormone	Gonadotropin-releasing hormone II receptor
V	10	Go02	O42329	Gonadotropin-releasing hormone	Gonadotropin-releasing hormone II receptor
VI	11	Ca01	Q98895	Cannabinoid	Cannabinoid receptor 1
VI	11	Ca01	Q71SP5	Cannabinoid	Cannabinoid receptor 1
VI	11	Ca01	P21554	Cannabinoid	Cannabinoid receptor 1
VI	11	Ca01	O02777	Cannabinoid	Cannabinoid receptor 1
VI	11	Ca01	P56971	Cannabinoid	Cannabinoid receptor 1
VI	11	Ca01	Q333S9	Cannabinoid	Cannabinoid receptor 1
VI	11	Ca01	Q801M1	Cannabinoid	Cannabinoid receptor 1
VI	11	Ca01	P47746	Cannabinoid	Cannabinoid receptor 1
VI	11	Ca01	Q5IS73	Cannabinoid	Cannabinoid receptor 1
VI	11	Ca01	Q9PUI7	Cannabinoid	Cannabinoid receptor 1A
VI	11	Ca01	Q98894	Cannabinoid	Cannabinoid receptor 1B
VI	11	Ca01	P20272	Cannabinoid	Cannabinoid receptor 1
VI	11	Ca02	Q9QZN9	Cannabinoid	Cannabinoid receptor 2
VI	11	Ca02	P47936	Cannabinoid	Cannabinoid receptor 2

VI	11	Ca02	P34972	Cannabinoid	Cannabinoid receptor 2
VII	13	Nu22	A6QLE7	Nucleotide-like	GPR52
VII	13	Nu22	Q9Y2T5	Nucleotide-like	GPR52
VII	13	Nu22	P0C5J4	Nucleotide-like	GPR52
VII	13	Nu29	Q99679	Nucleotide-like	GPR21
VII	13	Nu29	Q8BX79	Nucleotide-like	GPR21

Table S5. 197 GPCR sequences and their clustering of 47 groups predicted by MSClustering, N-J, and Louvain using the distance defined as E or $E^{0.02}$.

**Uniprot ID**	**MSC 1 [*E*, *E* 0.02]**	**N-J [$E^{0.02}$]**	**N-J [*E*]**	**Louvain [$E^{0.02}$]**	**Louvain [*E*]**
Q90X46	Nu17	1	2	3	2
B3DM66	Nu17	2	2	3	2
B2RPY5	Nu17	2	2	3	2
Q8N6U8	Nu17	2	2	3	2
Q2YDN1	Nu17	2	2	3	2
P79928	Nu07	3	2	2	3
Q9JJS7	Nu07	4	2	2	3
P51582	Nu07	4	2	2	3
O35811	Nu07	4	2	2	3
Q8BLG2	Nu28	5	2	2	3
Q99677	Nu28	5	2	2	3
Q8BMC0	Nu08	6	9	2	3
P43657	Nu08	6	9	2	3
Q4G072	Nu08	6	9	2	3
P32250	Nu08	6	20	2	3
P30543	Nu15	7	2	3	1
P29274	Nu15	7	2	3	2
P11617	Nu15	7	2	3	2
Q60613	Nu15	7	2	3	2
Q6TLI7	Nu15	7	2	3	2
P46616	Nu15	7	2	3	2
Q60614	Nu06	8	6	3	2
P29276	Nu06	8	31	3	2
P29275	Nu06	8	2	3	2
Q32ZE2	Nu06	8	2	3	2
Q1LZD0	Nu06	8	2	3	2
O13076	Nu06	8	2	3	2
Q6W3F4	Nu06	8	2	3	2
P49651	Nu14	9	10	2	3
P34996	Nu14	9	2	2	3
P49650	Nu14	9	10	2	3
P49652	Nu14	9	2	2	3
P47900	Nu14	9	10	2	3
P48042	Nu14	9	9	2	3
P59902	Nu14	9	10	2	3
P41232	Nu18	10	2	2	3
Q5YA25	Nu18	10	2	2	3
P41231	Nu18	10	2	2	3

P35383	Nu18	10	2	2	3
Q8BUD0	Nu16	11	2	2	3
Q4KLH9	Nu16	11	2	2	3
Q1JQB3	Nu16	11	2	2	3
P46093	Nu16	11	2	2	3
P50132	Nu16	11	2	2	3
Q8BFQ3	Nu20	12	2	2	3
O46685	Nu20	12	2	2	3
Q15743	Nu20	12	2	2	3
Q8BG55	Nu23	13	2	2	3
Q3ZBK9	Nu23	13	2	2	3
O14626	Nu23	13	2	2	3
Q9ESG6	Nu02	14	2	2	3
Q3SX17	Nu02	14	1	2	3
Q15391	Nu02	14	36	2	3
Q9CPV9	Nu19	15	2	2	3
Q9H244	Nu19	15	2	2	3
Q9EPX4	Nu19	15	2	2	3
Q95KC3	Nu19	15	2	2	3
Q9D8I2	Nu09	16	2	2	1
Q6GUG4	Nu09	16	2	2	3
Q62928	Pr08	17	2	1	1
Q62053	Pr08	17	12	1	1
P43116	Pr08	17	18	1	1
Q9XT82	Pr08	17	19	1	1
P79393	Pr07	18	2	1	1
P43119	Pr07	18	2	1	1
P43252	Pr07	18	2	1	1
P43253	Pr07	18	2	1	1
P28190	Nu05	19	6	3	2
P11616	Nu05	19	2	3	2
P49892	Nu05	19	2	3	2
P34970	Nu05	19	2	3	2
P47745	Nu05	19	2	3	2
Q5RF57	Nu05	19	2	3	2
Q60612	Nu05	19	2	3	2
P30542	Nu05	19	2	3	2
P25099	Nu05	19	6	3	2
Q61618	Nu26	20	2	3	2
Q0VC81	Nu01	20	2	3	2
O02667	Nu01	20	2	3	2
P33765	Nu01	20	2	3	2

P35342	Nu01	20	2	3	2
Q28309	Nu01	20	2	3	2
P28647	Nu26	20	2	3	2
Q98907	Nu25	21	2	2	3
Q9ERK9	Nu10	21	2	2	3
O93361	Nu25	21	2	2	3
Q63371	Nu10	21	2	2	3
Q15077	Nu10	21	2	2	3
Q6IYF8	Nu21	22	28	2	3
Q6Y1R5	Nu21	22	28	2	3
Q96P68	Nu21	22	37	2	3
Q98895	Ca01	23	2	3	1
Q71SP5	Ca01	23	2	3	1
P21554	Ca01	23	2	3	1
O02777	Ca01	23	2	3	1
P56971	Ca01	23	2	3	1
Q333S9	Ca01	23	2	4	4
Q801M1	Ca01	23	2	6	6
P47746	Ca01	23	2	7	7
Q5IS73	Ca01	23	2	8	8
Q9PUI7	Ca01	23	2	9	9
Q98894	Ca01	23	2	11	11
P20272	Ca01	23	2	12	12
Q9QZN9	Ca02	24	2	3	2
P47936	Ca02	24	35	5	5
P34972	Ca02	24	2	10	10
Q99MT7	Nu27	25	2	2	3
Q9BY21	Nu27	25	2	2	3
P35375	Pr01	26	43	1	1
P34995	Pr01	26	30	1	1
Q9BGL8	Pr01	26	23	1	1
P70597	Pr01	26	24	1	1
P34978	Pr10	27	26	1	1
Q95125	Pr02	27	42	1	1
P21731	Pr02	27	16	1	1
P30987	Pr10	27	27	1	1
P56486	Pr02	27	17	1	1
Q149R9	Nu03	28	2	2	3
Q3ZC80	Nu03	28	2	2	3
Q9H1C0	Nu03	28	2	2	3
O00398	Nu13	29	2	2	3
Q8BFU7	Nu13	29	2	2	3

P43088	Pr06	30	45	1	1
P37289	Pr06	30	25	1	1
P43117	Pr06	30	21	1	1
P43118	Pr06	30	22	1	1
Q28905	Pr06	30	25	1	1
Q61038	Nu12	31	47	2	3
Q8IYL9	Nu12	31	2	2	3
O35881	Nu02	32	29	2	3
Q9BPV8	Nu09	32	2	2	3
O35932	Pr09	33	34	1	1
A5D7K8	Pr03	33	33	1	1
Q13258	Pr03	33	46	1	1
Q9R261	Pr09	33	34	1	1
P70263	Pr09	33	38	1	1
O42329	Go02	34	44	31	31
Q01776	Go01	35	2	20	20
Q9MZI6	Go01	35	2	21	21
P30968	Go01	35	10	23	23
P32236	Go01	35	2	25	25
O18821	Go01	35	2	26	26
P32237	Go01	35	2	28	28
P49922	Go01	35	2	29	29
Q9TTI8	Go01	35	2	30	30
Q8CH60	Go01	35	2	32	32
Q19PY9	Go01	35	2	33	33
P30969	Go01	35	40	34	34
P21556	Pl01	36	2	13	13
Q9TTY5	Pl01	36	2	14	14
Q62035	Pl01	36	2	15	15
Q9XSD4	Pl01	36	2	16	16
P25105	Pl01	36	2	17	17
Q9GK76	Pl01	36	2	18	18
P46002	Pl01	36	11	19	19
Q95MG6	Go02	37	14	22	22
Q95MH6	Go02	37	5	24	24
Q95JG1	Go02	37	5	27	27
P43115	Pr05	38	2	1	1
P50131	Pr05	38	2	1	1
P46069	Pr05	38	2	1	1
P34979	Pr05	38	41	1	1
P34980	Pr05	38	12	1	1
P30557	Pr05	38	2	1	1

Q99MT6	Nu04	39	2	2	3
Q6IYF9	Nu04	39	5	2	3
Q9BXA5	Nu04	39	13	2	3
O43193	Th01	40	2	36	36
Q99P50	Th01	41	2	37	37
O08725	Th01	41	2	35	35
A5A4L1	Th01	41	2	42	42
A5A4K9	Th01	41	2	43	43
Q92847	Th01	41	32	46	46
Q95254	Th01	41	2	47	47
P43114	Pr04	42	2	1	1
P32240	Pr04	42	2	1	1
Q95KZ0	Pr04	42	2	1	1
Q8MJ08	Pr04	42	2	1	1
P35408	Pr04	42	2	1	1
Q28691	Pr04	42	2	1	1
Q99679	Nu29	43	15	3	2
A6QLE7	Nu22	43	2	3	2
Q9Y2T5	Nu22	43	2	3	2
P0C5J4	Nu22	43	2	3	2
Q8BX79	Nu29	43	2	3	2
Q9UNW8	Nu11	44	39	2	3
Q9Z282	Nu11	44	2	2	3
Q99678	Nu24	45	2	2	3
Q8BYC4	Nu24	45	2	2	3
P21761	Th02	46	4	38	38
Q01717	Th02	46	3	39	39
O46639	Th02	46	4	40	40
P34981	Th02	46	4	41	41
O93603	Th02	46	4	44	44
Q28596	Th02	46	4	45	45
Q8BZL4	Nu30	47	7	3	2
Q99680	Nu30	47	8	3	2

III. Manual of the MSClustering app

MSClustering: A Cytoscape tool for multi-level clustering of complex networks

1. Introduction

MSClustering is a tool for multi-level clustering of complex networks and provides immediate visualization and analyses in the Cytoscape platform. After transforming the N^2-distance matrix into an N-list of shortest connections for a system of N nodes, MSClustering automatically groups the system hierarchically for several characteristic levels of resolution. It is proportional to the system size in the memory of storage and operations of the algorithm. For a system of 2500 nodes, MSClustering takes about 27 seconds to perform a hierarchical clustering of the system. The algorithm of this app is efficient (computational time is roughly linear in N) and has been successfully applied to study various systems in biology, scientometrics, literature analyses, and finance. It is now integrated with Cytoscape to provide immediate visualization and statistical analyses.

Clustering/classification is an important step in understanding the present diversity and past evolutionary history of a complex system. As many important real-world clustering/classification problems are intrinsically hierarchical, it is desired to develop an efficient clustering tool for the automated clustering of complex systems at various characteristic levels. We have developed the MSClustering app in Cytoscape for an automated, efficient, and hierarchical clustering of complex networks at various characteristic resolution levels. Here, we have demonstrated that the constructed MSC tree provides phylogenetic information for complex systems from a distance-based approach. An example is the clustering of a 46-coronavirus network in three characteristic levels as shown in the screenshots. The constructed MSC tree is consistent with the best phylogenetic models from the character-based approach using IQTREE.

2. Installation

- Installing Cytoscape: Download and install the latest version of Cytoscape from https://cytoscape.org/.
- Installing MSClustering: Open Cytoscape and select Apps > App Manager from the main menu, as shown in Figure M1. In the App Manager,

select MSClustering in the list of All Apps and click the Install button.

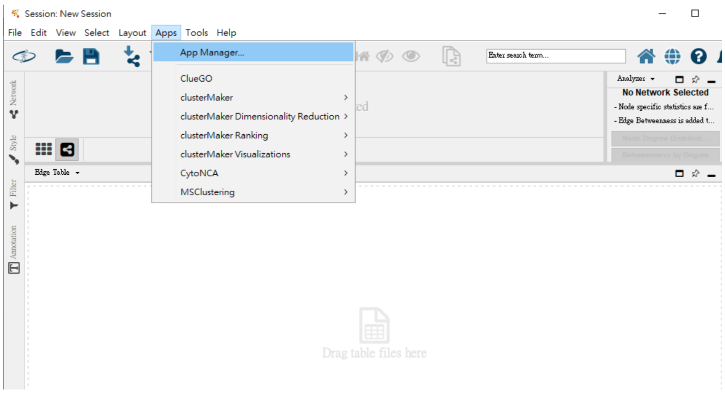

Figure M1. Installing MSClustering in Cytoscape.

3. Quick Tutorial

3.1 *Preparing Data Files*: Data files (distance matrix) could be in one of the following formats: .mat, .adj, .txt, .tsv, or .csv. As shown in Figure M2, the first row of the data file contains the outlier detection parameter (m) and the smallest number of groups at the highest level (N_{limit}), the second row contains nodes' ID, and the distance matrix is listed below the ID labels. A node is considered an outlier if its shortest distance to other nodes is larger than m times the median value of the shortest distances. To switch off these two options, just set m and N_{limit} to 0. Sample input files for a coronavirus network and a GPCR network can be downloaded from this link.

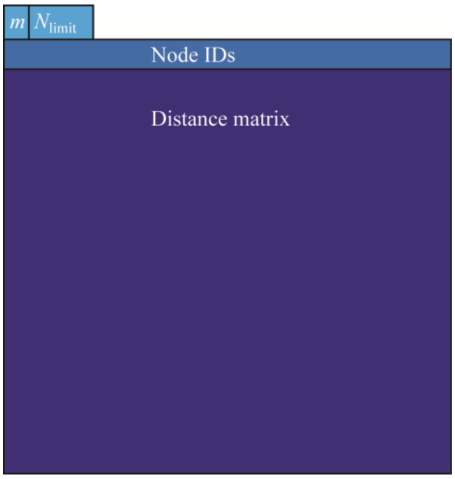

Figure M2. The format of the input data file. Here m is the parameter for outlier detection and N_{limit} is the smallest number of groups at the highest level of clustering.

3.2 *Importing a data file to the MSClustering*

- To create an MSC network go to the Cytoscape main menu and select

  Apps > MSClustering > Import Distance Matrix.

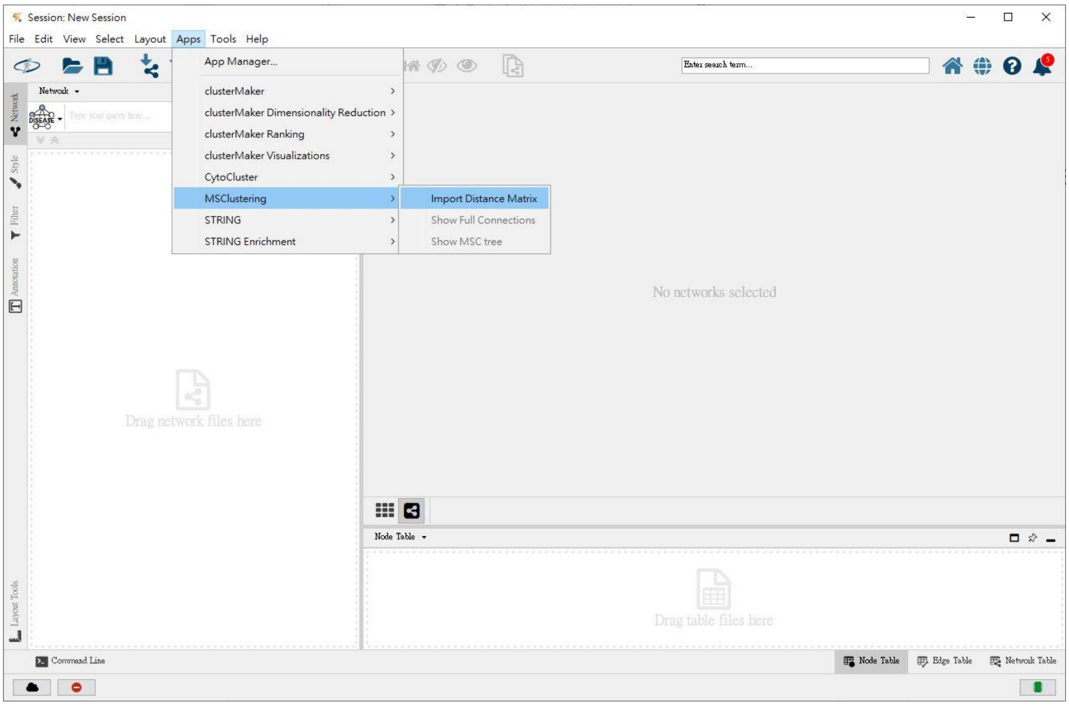

Figure M3. Running MSClustering by reading the input file.

3.3 *App manipulations*

- After importing the distance matrix, MSClustering will automatically cluster the system at various characteristic levels of resolution. Figure M4 shows a typical example consisting of three levels. There are three main areas in the app, including

1. Network panel, which lists all clustering levels of the network;

2. Network display, which displays the network diagram at the selected clustering level; and

3. Expression panel, which shows related data for all nodes and edges.

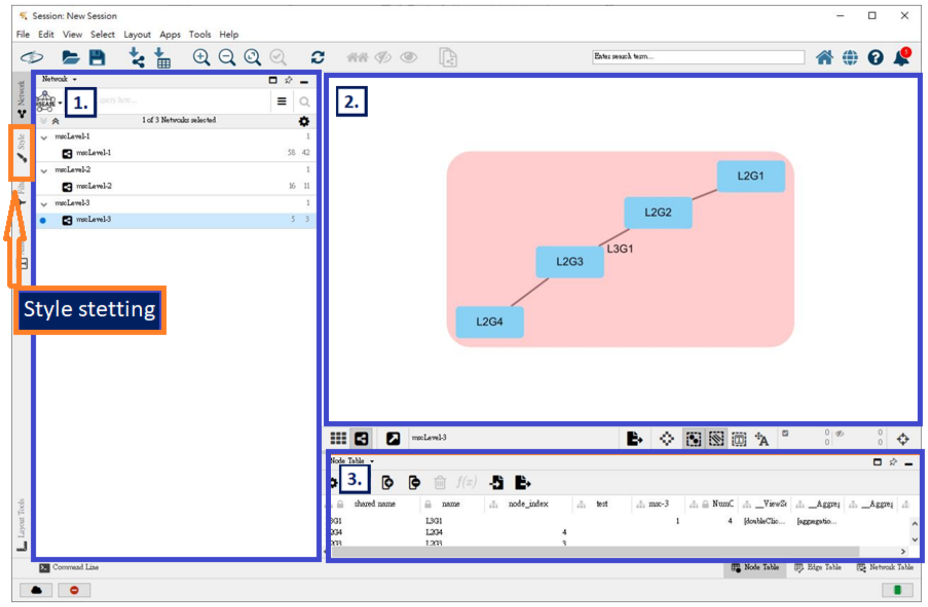

Figure M4.

- Style setting: This is the place to set the style details of the network figure, including 1. Change current style; 2. The setting of details; and 3. Switch for setting nodes, edges, or networks. For more information about Cytoscape styles, please visit http://manual.cytoscape.org/en/stable/Styles.html.

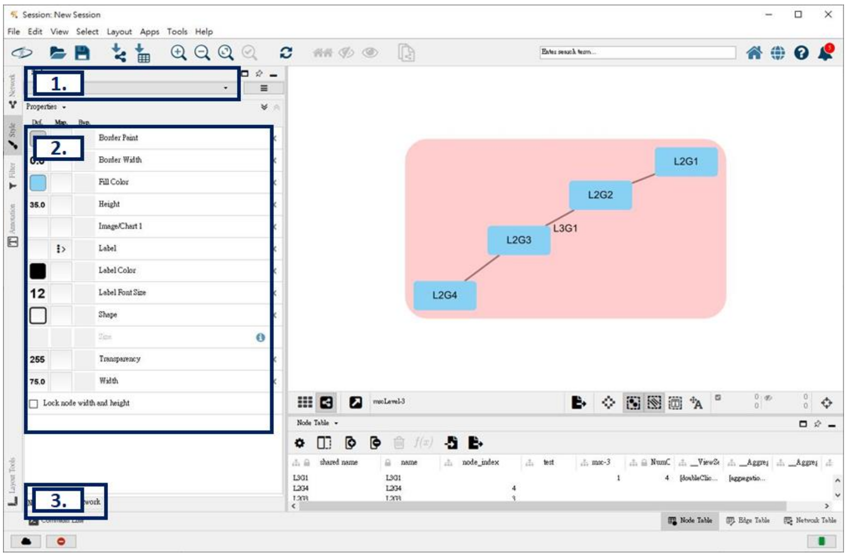

Figure M5.

- Building MSC trees and full connection trees: After clustering the system, the full-connection diagram and the MSC tree can be built from the app menu. Statistical analyses of the network can be performed by incorporating other Cytoscape apps (such as CytoNCA for centrality measures) with MSClustering. For biological networks, the

MSC tree is also a distance-based phylogenetic tree.

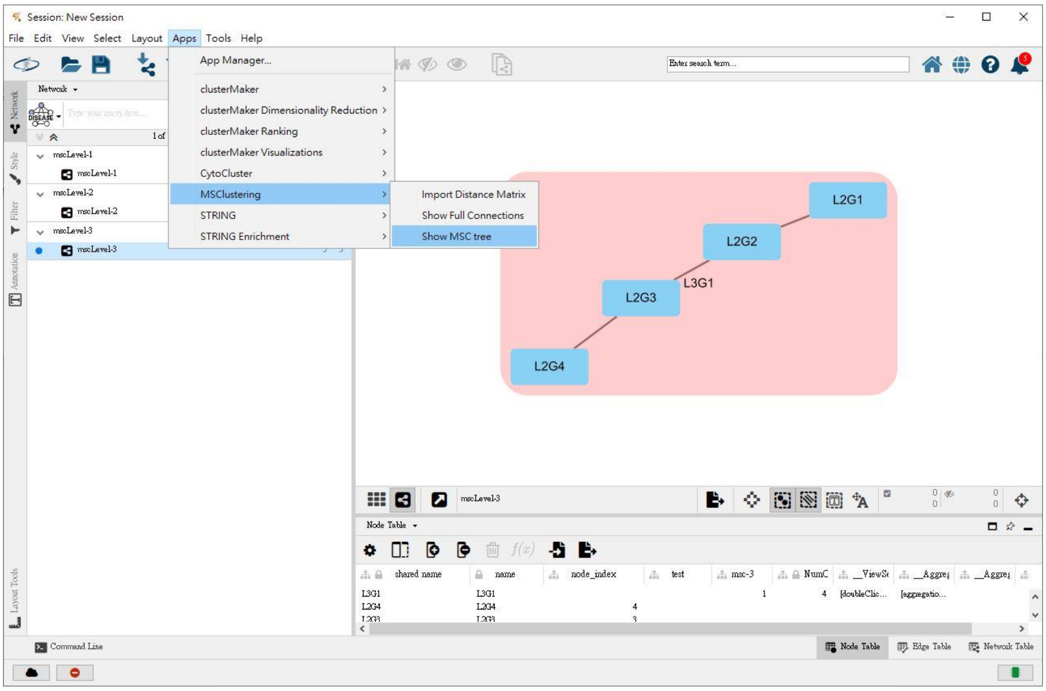

Figure M6.
